# Supplementary material for: Cytoplasmic circular dsDNA is a key constituent of stress granules
Source: bioRxiv. 2026 Mar 15:2026.03.12.711345. Preprint. [Version 1] doi: 10.64898/2026.03.12.711345 (PMC13060938; doi:10.64898/2026.03.12.711345)
Supplement: Supplement 1 [file media-1.pdf]

Supplementary Information

Cytoplasmic circular dsDNA is a key constituent of stress granules

Natalia A. Demeshkina[1*] & Adrian R. Ferré-D'Amaré[1*]

[1]Laboratory of Nucleic Acids, National Heart, Lung, and Blood Institute, National Institutes of Health, Bethesda, MD, 20892, USA

*Corresponding authors: natalia.demeshkina@nih.gov or adrian.ferre@nih.gov

Supplementary Figures

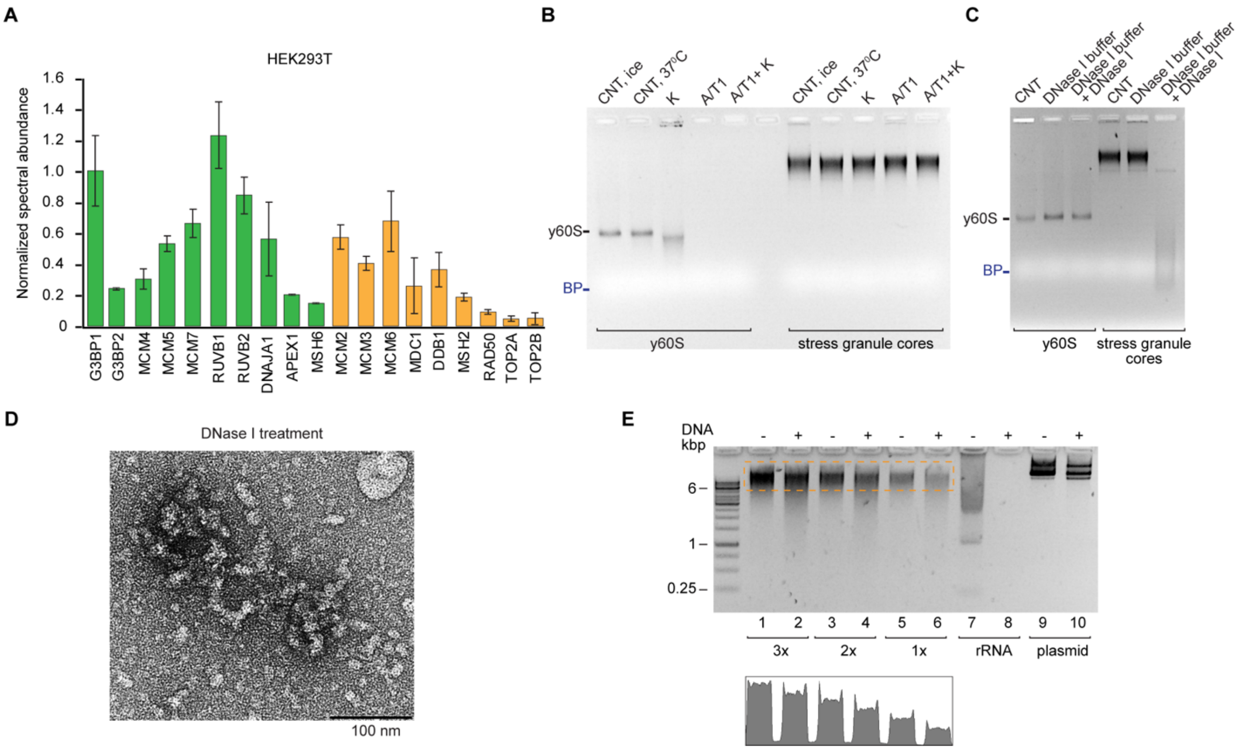

Figure S1. Proteinaceous and DNA constituents of stress garnule cores, related to Figure 1.

(A) Abundance of representative DNA-binding proteins in the proteomes of stress granule cores isolated from early-log HEK293T cells[1]. Green color denotes DNA-binders also found in immunoprecipitated stress granules from late-log U-2 OS cells[2]; orange color marks DNA-binders additionally identified in the cores of HEK293T cells. Relative abundance of proteins between datasets was normalized using G3BP1 (data are mean ± s.d. from n = 3 biological replicates).

(B) Native agarose gel electrophoresis (ethidium bromide stain) of the yeast large ribosomal subunit (y60S) and yeast stress granule cores treated with proteinase K, ribonucleases A and T1 (A/T1), and combination of these enzymes. Incubation was performed at 37 °C for 24 hours.

(C) Native agarose gel electrophoresis (ethidium bromide stain) of y60S and yeast stress granule cores treated with DNase I. Incubation was performed at 37 °C for 1 hour.

(D) Negative-stain electron micrograph of yeast stress granule cores treated with DNase I for 30 minutes at 30 °C. Magnification (× 13,000).

(E) Alkaline hydrolysis of total nucleic acids extracted from yeast stress granule cores (15, 10 and 5 ng), rRNA (15 ng) and 11 kbp plasmid (15 ng), analyzed by non-denaturing agarose gel electrophoresis (SYBR Gold stain). Samples marked (+) were incubated with 50 mM KOH for 15 min at 95 °C. Bottom graph, integrated signal for the dashed box. In (B) and (C), BP stands for bromophenol blue.

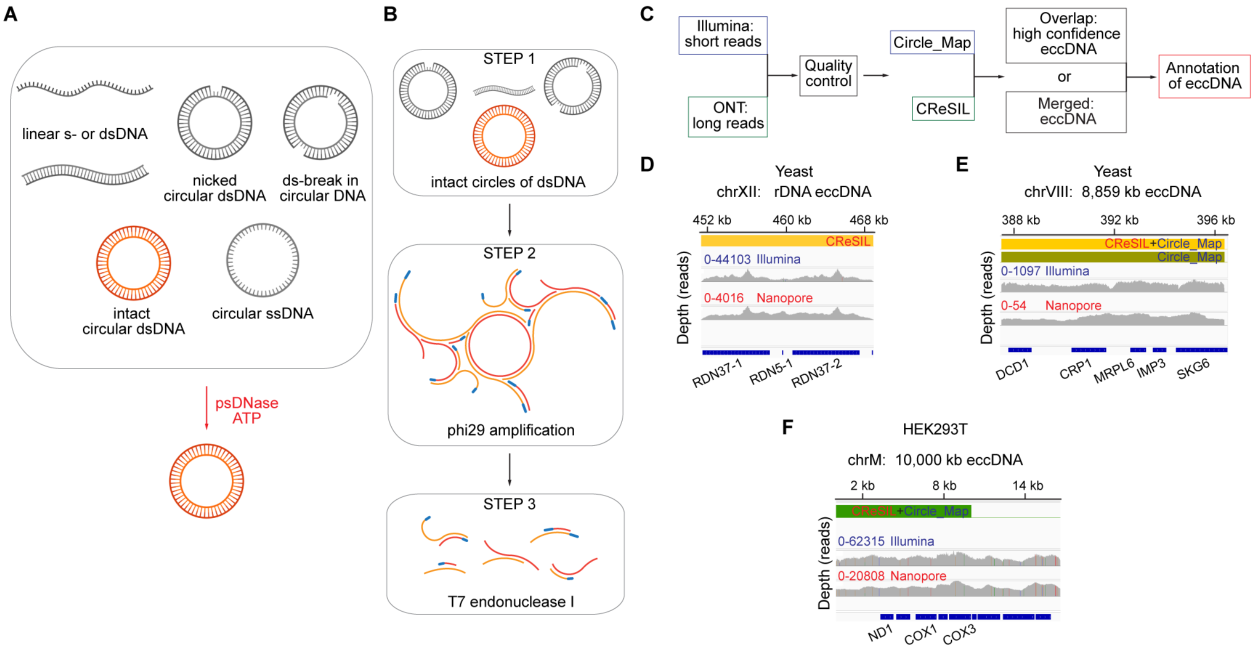

Figure S2. Workflow for analysis of DNA from stress granule cores by eccDNA assemblers, related to Figure 2.

(A) Substrate specificity of psDNase. Species of DNA hydrolyzed by psDNase in the presence of ATP are in black.

(B) Main steps of modified Circle-Seq treatment applied to enrich (step 1), amplify (step 2) and debranch (step 3) circular DNA from stress granule cores for further sequencing.

(C) Bioinformatic workflow of processing DNA reads for assembly into eccDNA circles.

(D-F) Examples of genomic regions covered by short (Illumina) and long (Nanopore; ONT) reads, whose sequences are assembled in eccDNAs for yeast (D) and (E) and human (F) stress granule cores. Panels (A) and (B) created with BioRender.com.

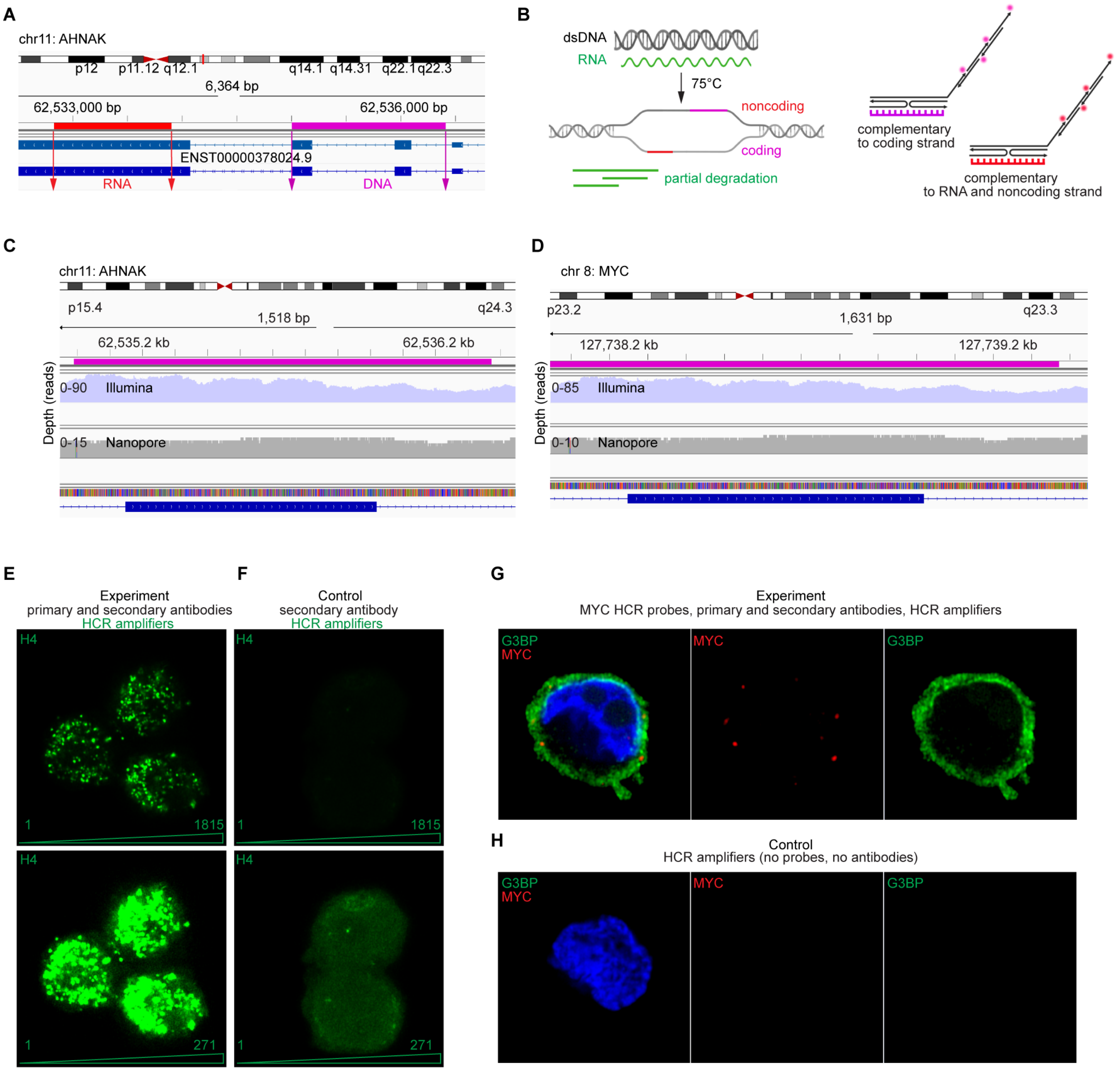

Figure S3. Detection of stress granule core constituents in HEK293T cells by HCR fluorescence, related to Figure 3.

(A) Exemplary design of FISH probes for targeting DNA (magenta) and RNA (red) counterparts of a gene of interest.

(B) Schematic of heat-denaturation step of HCR-FISH protocol aimed to detect DNA coding region (magenta). Concurrent detection of transcribed RNA with corresponding region of non-coding DNA strand is indicated (red).

(C-D) Representative DNA Illumina and Nanopore reads covering regions within AHNAK (C) and MYC (D) genes to which HCR-FISH probes (magenta) were designed.

(E-F) Abundance of histone H4 in the cytoplasm of HEK293T cells. Two gains (top and bottom) demonstrate sensitivity and specificity of HCR immunolabeling.

(G-H) HCR amplifiers are highly specific to primary targets (FISH probes or antibodies).
In (E-H) all images represent one middle Airyscan confocal Z-stack of 0.2 μm. Nuclei were stained by Hoechst 33342 (blue) in (G) and (H).

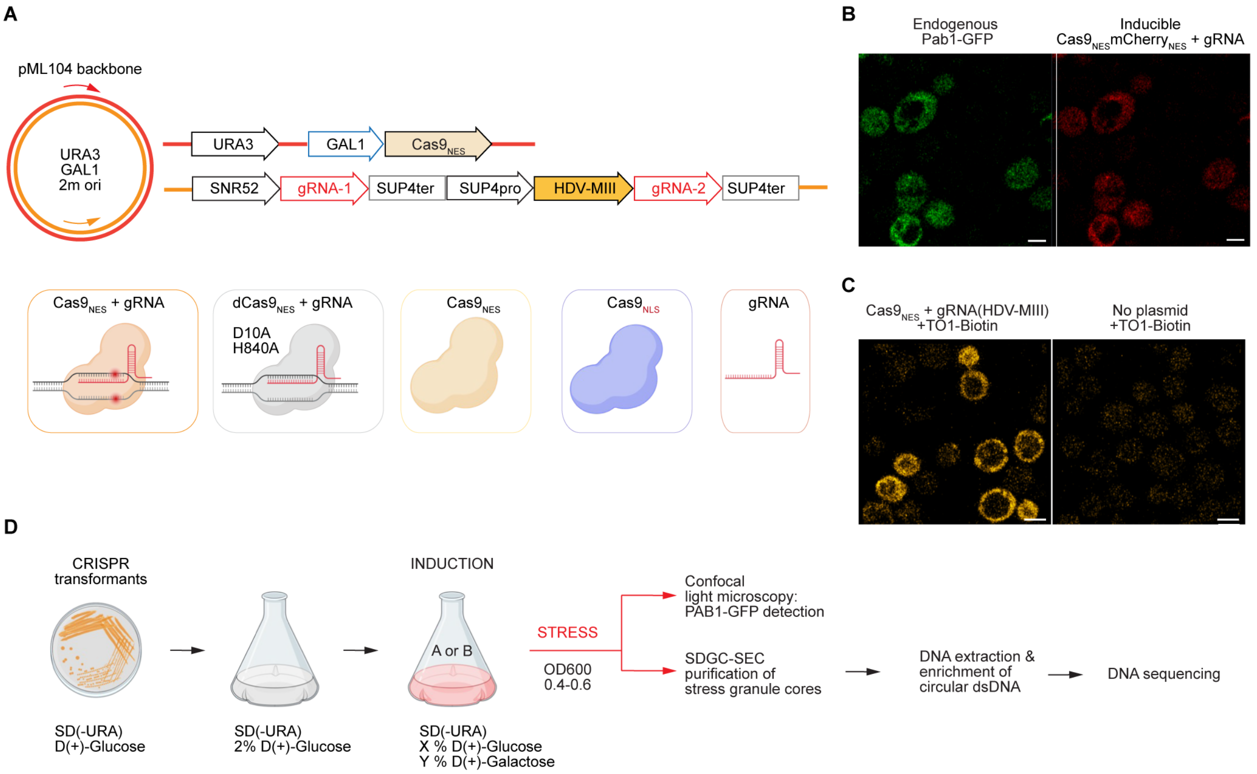

Figure S4. Design of CRISPR experiments for targeting cytoplasmic eccDNA in yeast stress granule cores, related to Figure 4.

(A) Major regulatory and coding motifs of CRISPR elements in modified vector pML104. Botton panel displays experimental variants of yeast transformants with CRISPR-pML104. NES, nuclear export signal; NLS, nuclear localization signal.

(B) Transiently expressed $Cas9_{NES}mCherry_{NES}$ localizes to cytosol together with PAB1-GFP (condition A, see (D), no stress).

(C) Detection of gRNA in yeast cytoplasm using fluorophore TO1-Biotin and fluorogenic RNA aptamer Mango-III(A10U) (see Methods) encoded in HDV ribozyme at 5'-end of gRNA-2 (see (A)). The experiments were conducted with strain JD1370 free from endogenous or transient fluorescent fusions.

(D) Growth steps of CRISPR-pML104 transformants with indication of major alterations in media, viz. changing D(+)-glucose to D(+)-galactose ratio to induce expression of Cas9 and addition of sodium azide to stress cells. Condition A: 0.2% w/v D-(+)-glucose and 2% w/v D-(+)-galactose for 4 h. Condition B: 1.5% w/v D-(+)-glucose and 0.75% w/v D-(+)-galactose, overnight.

In (B) and (C), Airyscan images of middle Z-stack plane (0.14 μm). Scale bar 2 μm. Panels (A) and (D) created with BioRender.com.

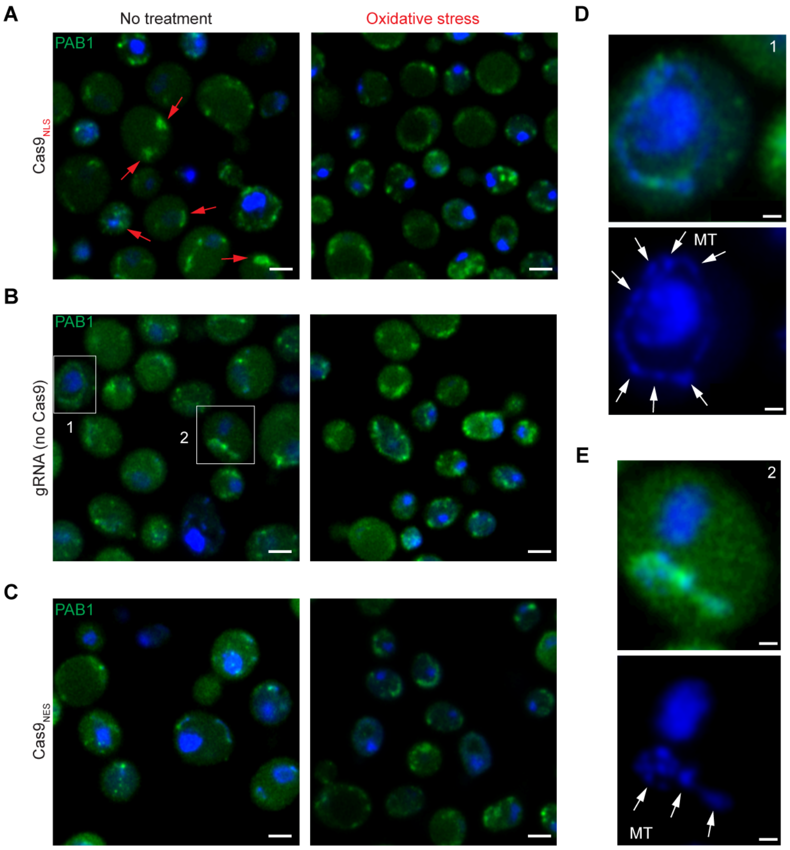

Figure S5. Phenotypic changes of *S. cerevisiae* in response to transient expression of individual CRISPR elements, related to Figures 4 and 5.

(A-C) Confocal microscopy of *S. cerevisiae* with endogenous PAB1-GFP (green) and transiently expressed Cas9 (GAL promoter) or gRNAs (see Figure S4A). Treatment conditions and nuclear (NLS) or cytoplasmic (NES) tags are indicated. In (A), arrows (red) indicate accumulation of PAB1-GFP signal observed in the absence of stress.

(D-E) Colocalization of PAB1-GFP with mitochondria in the absence of stress (framed in (B)). Upper images (488 and 405 nm channels) are split into individual channels to mark mitochondria (lower panels; arrows).

All images represent one middle Airyscan confocal Z-stack of 0.14 µm with nuclei and mitochondria stained by DAPI (blue). Scale bars 2 µm in (A-C) and 0.5 µm in (D) and (E).

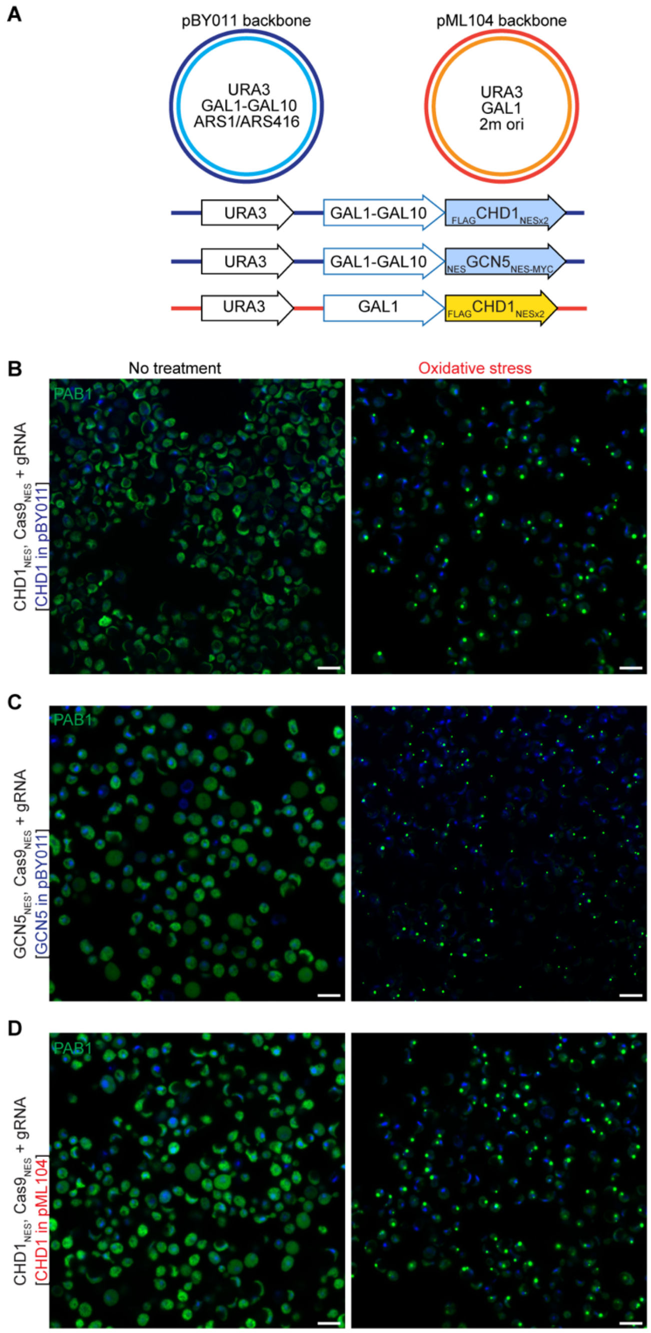

Figure S6. Chromatin binders CHD1 and GCN5 compete out CRISPR and restore stress granule formation in cytoplasm, related to Figures 4 and 5.

(A) Schematics of pBY011 and pML104 main elements used to express chromatin remodeler CHD1 or histone acetyltransferase GCN5.

(B) Transient co-expression of cytoplasmic CHD1 encoded into pBY011 backbone with cytoplasmic CRISPR preserves canonical stress response.

(C) Transient co-expression of cytoplasmic GCN5 encoded into pBY011 backbone with cytoplasmic CRISPR preserves canonical stress response.

(D) Transient co-expression of cytoplasmic CHD1encoded into pML104 backbone with cytoplasmic CRISPR preserves canonical stress response.

Right panels in (B) to (D), cells treated with 0.5% w/v sodium azide (Methods). All images represent one middle Airyscan confocal Z-stack of 0.14-0.17 μm with nuclei and mitochondria stained by DAPI (blue). Scale bar 5 μm.

Supplementary Tables

Table S1. Antibodies used in IF and HCR IF light microscopy imaging of HEK293T cells.

Primary Antibody [dilution]	Secondary Antibody [dilution]	Amplifier
Anti-VCP, mouse, monoclonal [1:100] (Santa Cruz Biotechnology, Cat# sc-57492)	Anti-mouse, donkey [1:500], B5 initiator Molecular Instruments, Inc.	B5-647
Anti-histone H4, rabbit, monoclonal [1:70 – 1:100] (Abcam, Cat# ab177840)	Anti-rabbit, donkey [1:500], B1 initiator Molecular Instruments, Inc.	B1-488
Anti-histone H3.1, rabbit, monoclonal [1:30 to 1:90] (Novus Biologicals, Cat# NBP3-26228)	Anti-rabbit, donkey [1:500], B1 initiator Molecular Instruments, Inc.	B1-488
Anti-G3BP, mouse, monoclonal [1:200] (Abcam, Cat# ab56574)	Anti-mouse, donkey [1:500], B5 initiator Molecular Instruments, Inc.	B5-647
Anti Lamin A + Lamin C, rabbit, monoclonal [1:400] (Abcam, Cat# ab108595)	Anti-rabbit, donkey [1:800], B1 initiator Molecular Instruments, Inc.	B1-488
Anti-Caprin1, rabbit, polyclonal [1:400] (Abcam, Cat# ab244360)	Anti-rabbit, donkey [1:750], B4 initiator Molecular Instruments, Inc.	B4-546
Anti-TOMM20, rabbit, monoclonal [1:250] (Abcam, Cat# ab186735)	Anti-rabbit, donkey [1:500], B1 initiator Molecular Instruments, Inc.	B1-488
Anti-DNA, mouse, monoclonal [1:200] (Thermo Fisher Scientific, Cat # 690014S)	Anti-mouse, goat [1:800], Alexa Fluor 568 (Invitrogen, Cat# A11004)	N/A

Table S2. Sequence Read Archive deposition summary for raw DNA sequences.

Bioproject	Accession	Species	Sequencing platform	Sample name
PRJNA1306188	SRR34996475	*S. cerevisiae*	Illumina	Cas9nes_gRNA_01
PRJNA1306188	SRR34996474	*S. cerevisiae*	Illumina	Cas9nes_gRNA_02
PRJNA1306188	SRR34996473	*S. cerevisiae*	Illumina	dCas9nes_gRNA_01
PRJNA1306188	SRR34996472	*S. cerevisiae*	Illumina	dCas9nes_gRNA_02
PRJNA1306188	SRR34996471	*S. cerevisiae*	Illumina	No_tranform_01
PRJNA1306188	SRR34996470	*S. cerevisiae*	Illumina	No_tranform_02
PRJNA1306188	SRR34996469	*S. cerevisiae*	Illumina	Cas9nes_01
PRJNA1306188	SRR34996468	*S. cerevisiae*	Illumina	Cas9nes_02
PRJNA1306188	SRR35009179	*S. cerevisiae*	Illumina	Cas9nes_gRNA_CHD1nes_01
PRJNA1306188	SRR35009178	*S. cerevisiae*	Illumina	Cas9nes_gRNA_CHD1nes_02
PRJNA1306188	SRR35009463	*S. cerevisiae*	Illumina	ySCGs_Illumina_01
PRJNA1306188	SRR35009462	*S. cerevisiae*	Illumina	ySCGs_Illumina_02
PRJNA1306188	SRR35009461	*S. cerevisiae*	ONT	ySGCs_ONT_01
PRJNA1306188	SRR35009460	*S. cerevisiae*	ONT	ySGCs_ONT_02
PRJNA1305524	SRR35178678	*H. sapiens*	ONT	7E_Dna_nanopore
PRJNA1305524	SRR35178677	*H. sapiens*	ONT	8E_Dna_nanopore
PRJNA1305524	SRR35178676	*H. sapiens*	ONT	9E_Dna_nanopore
PRJNA1305524	SRR35178675	*H. sapiens*	Illumina	7E_Dna_Illumina
PRJNA1305524	SRR35178674	*H. sapiens*	Illumina	8E_Dna_Illumina
PRJNA1305524	SRR35178673	*H. sapiens*	Illumina	9E_Dna_Illumina
PRJNA1305524	SRR34981497	*H. sapiens*	ONT	hSGCs 1_early_ONT
PRJNA1305524	SRR34981496	*H. sapiens*	ONT	hSGCs 2_late_ONT
PRJNA1305524	SRR34981495	*H. sapiens*	ONT	hSGCs 3_early_ONT
PRJNA1305524	SRR34981494	*H. sapiens*	ONT	hSGCs 4_late_ONT
PRJNA1305524	SRR34981493	*H. sapiens*	ONT	hSGCs 5_early_ONT
PRJNA1305524	SRR34981492	*H. sapiens*	ONT	hSGCs 6_late_ONT
PRJNA1305524	SRR35328255	*H. sapiens*	Illumina	hSGCs 1_early_Illumina
PRJNA1305524	SRR35328254	*H. sapiens*	Illumina	hSGCs 2_late_Illumina
PRJNA1305524	SRR35328253	*H. sapiens*	Illumina	hSGCs 3_early_Illumina
PRJNA1305524	SRR35328252	*H. sapiens*	Illumina	hSGCs 4_late_Illumina
PRJNA1305524	SRR35328251	*H. sapiens*	Illumina	hSGCs 5_early_Illumina
PRJNA1305524	SRR35328250	*H. sapiens*	Illumina	hSGCs 6_late_Illumina

Table S3. Cloning materials for CRISPR targeting cytoplasmic eccDNA with Ty1 in yeast.

Plasmid Name	Description
pML104	Original (Addgene #67638)<br>GAP-$Cas9_{NLS}$<br>Scaffold (tracrRNA) under SNR52<br>No crRNA
pML104-GAL1-$Cas9_{NES}$-Scaffold	GAL1-$Cas9_{NES}$<br>Scaffold RNA (tracrRNA) / SNR52<br>No crRNA
pML104-GAL1-$Cas9_{NES}$	GAL1-Cas9-NES<br>No scaffold RNA<br>No crRNA
pML104-GAL1-$Cas9_{NES}$-Ty1	GAL1-Cas9-NES-Ty1<br>crRNA-1: SNR52-Ty11<br>crRNA-2: SUP4-$Ty12^{HDV}$ (minus MIII insert)
pML104-GAL1-$Cas9_{NES}$-Ty1-MIII	GAL1-Cas9-NES-Ty1<br>crRNA-1: SNR52-Ty11<br>crRNA-2: SUP4-$Ty12^{HDV\text{-}MIII}$ (plus MIII insert)
pML104-GAL1-$dCas9_{NES}$-Ty1	GAL1-dCas9-NES-Ty1<br>crRNA-1: SNR52-Ty11<br>crRNA-2: SUP4-$Ty12^{HDV}$
pML104-GAL1-$Cas9_{NES}$-$mCherry_{NES}$-Ty1-MIII	GAL1-Cas9-NES-mCherry-NES-Ty1<br>crRNA-1: SNR52-Ty11<br>crRNA-2: SUP4-$Ty12^{HDV\text{-}MIII}$ (plus MIII insert)

Table S4. Primers and fragments designed for modification of the pML104 backbone.

Name	Sequence
NES-For	5’- cagatttccgagttttctaaacgcgtcattctcgctgat
NES-Rev	5’- tgccaatctaaacgataccacggccgctctagagaaatgg
GAL1-For	5’- gtatcgataagcttgatatcgaattcagtctactagtcagttcgagtttacg
GAL1-Rev	5’- tttctcgtgataggccacctcgtcgacgatattaccgaagatgg
CON-SNR52	5’-<br>ggccgtggtatcgtttagattggcaattacagtgtcttagctcacatgcttataactaattacatgactcgaagac<br>ataaaaaacaaaaaaagatcatttatctttcactgcggagaagtttcgaacgccgaaacatgcgcaccaacttt<br>cacttctacagcgtttgaccaaaatcttttgaacagaacattgtagggtgtgaaaaaatgcgcacctttaccg
CON-For	5’- cgagcaaatgcctgcaaatcgctccccatttctctagagcggccgtggtatcgtttagattgg
CON-Rev	5’- gaagtacaactctagattttgtagtgccctcttgggctagcggtaaaggtgcgcattttttcac
MIII(U10A)	5’-GTACGAAGGAAGGTTTGGTATGGGGTAGTTGTCGTAC-3’
Ty1-2-MIII-HDV	5’-<br>agacataaaaaacaaaaaaagcaccgactcggtgccactttttcaagttgataacggactagccttattttaact<br>tgctatttctagctctaaaacGGTCTTTATATAGACCAGGAaaa**gtcccattcgccacc**GT<br>ACGACAACTACCCCATACCAAACCTTCCTTCGTAC**ggtgttgcccagccgg**<br>**cgccagcgaggaggctgggaccatgccgg**ccatctctcccgggggcgagtcgaacgcccgatctcaag<br>atttcgtagtgataaattacagtcttgcgccttaaaccaacttggctaccgagagtatttaattgttgaagaaaga<br>gtatactacataacacatata -3’
Ty12-For	5’- gcatgaggtcgctcttattgaccacacctctaccggcatgagacataaaaaacaaaaaaagcaccgact
Ty11-For	5’- gatcg**accaggtctttatatagacc**-3’
Ty11-Rev	5’- **ggtctatataaagacctggt**c-3’
Ty12-Rev	5’-<br>tgagctaagacactgtaattgccaatctaaacgataccactatatgtgttatgtagtatactctttcttcaacaatta<br>aatactctcg
a29c-s	5'-gtattctatcggactggccatcgggactaatagcg-3'
a29c-as	5'-cgctattagtcccgatggccagtccgatagaatac-3'
c2518g-a2519c-s	5'-ctctgagggacgatggcgtccacgtcgtagtc-3'
c2518g-a2519c-as	5'-gactacgacgtggacgccatcgtccctcagag-3'

Table S5. Addgene deposit IDs for plasmids used in the study.

Name	Sequence ID [deposit]
pML104_Gal1_Cas9NES_Ty1MIIIA10U	215655 [83841]
pML104_Gal1Cas9NES_Ty1	215656 [83841]
pML104_Gal1dCas9NES_Ty1	215658 [83841]
pML104-Gal1Cas9NES	215659 [83841]
pBY011_Flag_CHD1_NES2	215660 [83841]
pML104_Ty1_11_12	246009 [86470]
pML104_Gal1_Cas9NLS	246012 [86470]
pML104_Gal1_Flag_CHD1NES2	246015 [86470]
pBY011_NES_GCN5_NES_MYC	246016 [86470]

Supplementary References

1. Demeshkina, N.A., and Ferre-D'Amare, A.R. (2025). Large-scale purifications reveal yeast and human stress granule cores are heterogeneous particles with complex transcriptomes and proteomes. Cell Rep *44*, 115738. 10.1016/j.celrep.2025.115738.

2. Jain, S., Wheeler, J.R., Walters, R.W., Agrawal, A., Barsic, A., and Parker, R. (2016). ATPase-Modulated Stress Granules Contain a Diverse Proteome and Substructure. Cell *164*, 487-498. 10.1016/j.cell.2015.12.038.
